# A222 EFFECT OF CLINICAL FACTORS ON IBD TREATMENT RESPONSE: FINDINGS FROM A NOVEL SINGLE CENTER PATIENT REGISTRY

**DOI:** 10.1093/jcag/gwac036.222

**Published:** 2023-03-07

**Authors:** C Lavalle, R Khanna, S Asfaha

**Affiliations:** Gastroenterology, University of Western Ontario, London, Canada

## Abstract

**Background:**

Inflammatory bowel disease (IBD), comprising both Crohn’s disease as well as ulcerative colitis, has shown heterogenous response to therapy. Over the past decades, targeted biologic therapies have become the mainstay of treatment. Even with the rapid pace of progress in this field, roughly a third of patients do not show an initial response to these treatments. The ability to accurately predict treatment response to therapy would both improve patient safety and satisfaction as well as decrease costs from ineffective therapies.

**Purpose:**

The primary aim of this project is to identify clinical factors predictive of disease response to induction biologic therapy in inflammatory bowel disease through the creation of a novel single center patient registry.

**Method:**

Initially, a single center tissue biopsy registry of IBD patients receiving colonoscopy was created. Retrospective clinical data was subsequently collected on patients identified to have initiated or changed biologic therapy after entry into the registry. Clinical data regarding age, sex, comorbidities, lifestyle factors, time since initial diagnosis, extra intestinal manifestations, serum markers, prior IBD treatments, planned treatment, clinical response, as well as endoscopic response were collected. A Pilot study of the first 30 patients identified was undertaken. Individual clinical factors were compared between patients with a documented clinical response to biologic therapy and non-responders at the group level as well as by biologic therapy initiated.

**Result(s):**

No statistically significant differences in collected clinical data parameters were observed between treatment responders and non responders in aggregate as well as when sub selected by individual biologic therapy initiated, bio-naive status, and steroid dependence.

**Conclusion(s):**

This project has demonstrated that clinical factors alone do not adequately predict disease response in inflammatory bowel disease. Future work will expand this data registry to further clinical factors and continue patient enrolment. Next steps also include the addition of tissue level data, in particular tissue level RNA data. It is hoped that these parameters, either individually or in combination, will provide a robust and accessible predictor of response to biologic therapy in inflammatory bowel disease.

**Please acknowledge all funding agencies by checking the applicable boxes below:**

Other

**Please indicate your source of funding below:**

Western University Department of Medicine

**Disclosure of Interest:**

None Declared

